# Roles of octopamine and dopamine in appetitive and aversive memory acquisition studied in olfactory conditioning of maxillary palpi extension response in crickets

**DOI:** 10.3389/fnbeh.2015.00230

**Published:** 2015-09-01

**Authors:** Yukihisa Matsumoto, Chihiro-Sato Matsumoto, Ryo Wakuda, Saori Ichihara, Makoto Mizunami

**Affiliations:** ^1^Faculty of Science, Hokkaido UniversitySapporo, Japan; ^2^Faculty of Liberal Arts, Tokyo Medical and Dental UniversityIchikawa, Japan; ^3^Graduate School of Life Science, Hokkaido UniversitySapporo, Japan

**Keywords:** classical conditioning, memory acquisition, dopamine, octopamine, appetite learning, aversive learning, *Gryllus bimaculatus*, insects

## Abstract

Elucidation of reinforcing mechanisms for associative learning is an important subject in neuroscience. Based on results of our previous pharmacological studies in crickets, we suggested that octopamine and dopamine mediate reward and punishment signals, respectively, in associative learning. In fruit-flies, however, it was concluded that dopamine mediates both appetitive and aversive reinforcement, which differs from our suggestion in crickets. In our previous studies, the effect of conditioning was tested at 30 min after training or later, due to limitations of our experimental procedures, and thus the possibility that octopamine and dopamine were not needed for initial acquisition of learning was not ruled out. In this study we first established a conditioning procedure to enable us to evaluate acquisition performance in crickets. Crickets extended their maxillary palpi and vigorously swung them when they perceived some odors, and we found that crickets that received pairing of an odor with water reward or sodium chloride punishment exhibited an increase or decrease in percentages of maxillary palpi extension responses to the odor. Using this procedure, we found that octopamine and dopamine receptor antagonists impair acquisition of appetitive and aversive learning, respectively. This finding suggests that neurotransmitters mediating appetitive reinforcement differ in crickets and fruit-flies.

## Introduction

Associative learning provides animals with the ability to adapt their behavior to a change in the environment. Elucidation of reinforcing mechanisms for associative learning is an important subject in neuroscience. In mammals, there is strong evidence that midbrain dopamine neurons mediate appetitive reinforcement signals (Schultz, 2006, 2013), and results of some studies have suggested that they also mediate aversive reinforcement signals (Matsumoto and Hikosaka, 2009). In crickets, we obtained pharmacological evidence suggesting that octopamine receptor antagonists (epinastine and mianserin) impair appetitive learning but not aversive learning, whereas dopamine receptor antagonists (flupenthixol, fluphenazine, chlorpromazine, and spiperone) impair aversive learning but not appetitive learning, and we thus proposed that octopamine and dopamine neurons mediate reward and punishment, respectively, in associative learning (Unoki et al., 2005, 2006; Mizunami et al., 2009; Nakatani et al., 2009). This is in accordance with suggestions from pharmacological studies on honey bees that octopamine participates in appetitive learning with sucrose reward (Hammer and Menzel, 1998) and dopamine participates in aversive learning with electric shock punishment (Vergoz et al., 2007). Based on results of recent studies using transgenic fruit-flies, however, it was concluded that different sets of dopamine neurons mediate appetitive and aversive reinforcements, while octopamine neurons transmit sweet taste signals to dopamine neurons in appetitive learning with sugar reward (Kim et al., 2007; Selcho et al., 2009; Burke et al., 2012; Liu et al., 2012; Lin et al., 2014). These findings in fruit-flies differ from those in crickets and urge us to re-examine the validity of our suggestions.

It should be pointed out that tests on the effects of conditioning in our studies in crickets were preformed 30 min after training or later, due to limitations of our previous “classical conditioning and operant testing” procedure (Matsumoto and Mizunami, 2002; Unoki et al., 2005), and thus the possibility that octopamine and dopamine are needed for 30 min memory retention but not for initial acquisition of learning was not ruled out. In our previous studies, crickets that received conditioning trials were allowed to rest for at least 15 min in the conditioning beaker before being transferred to the test apparatus because we speculated that disturbance immediately after conditioning may disrupt consolidation of memory. Moreover, after transferring the crickets to the test arena, crickets were first placed in a waiting chamber for at least 4 min for acclimation. Thus, we were not able to test acquisition performance immediately after training. This is in contrast to studies in flies in which the effect of conditioning was tested immediately after training. Therefore, it is critically important to establish procedures to allow investigation of possible roles of octopamine and dopamine in initial acquisition of learning in crickets.

In this study, we first established a conditioning procedure to allow evaluation of acquisition performance in crickets. We observed that crickets extended their maxillary palpi and vigorously swung them when water was applied to the antennae, which we refer to as maxillary palpi extension responses (MERs). Some odors such as vanilla and maple odors easily evoked MERs, while other odors such as peppermint and apple odors rarely induced MERs. In this study, we found that the MER to an odor is increased by pairing of the odor with water reward. This is analogous to olfactory conditioning of proboscis extension responses (PER) in honey bees, in which pairing of an odor and sucrose reward leads to an increase of the PER (Menzel and Giurfa, 2006; Giurfa, 2007; Menzel, 2012; Matsumoto et al., 2014). Moreover, we found that crickets exhibit a decrease in the MER to some odors by pairing of the odors with sodium chloride punishment, thus allowing the study of appetitive conditioning and aversive conditioning in a very similar experimental situation. We used these procedures to determine whether octopamine and dopamine are indeed required for acquisition of appetitive and aversive learning in crickets.

## Materials and methods

### Insects

Adult male crickets, *Gryllus bimaculatus*, at 1–2 weeks after the imaginal molt were used. They were reared in a 12 h: 12 h light: dark cycle at 27 ± 2°C and were fed a diet of insect pellets and water *ad libitum*. Three days before the start of the experiment, crickets were individually placed in 100 ml glass beakers and fed a diet of insect pellets *ad libitum* but were deprived of drinking water to enhance their motivation to search for water.

### Conditioning

**Figure 1:**
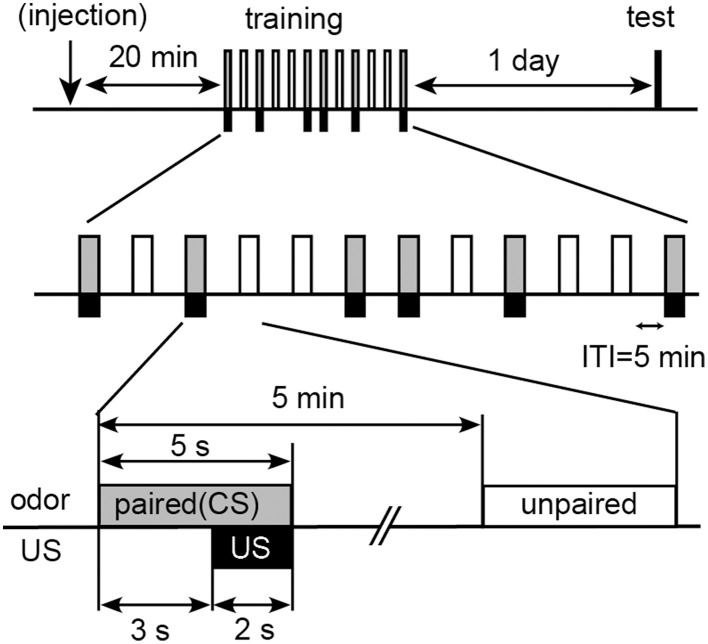
**Procedures for differential olfactory conditioning with water reward or sodium chloride punishment**. Crickets were subjected to presentation of one odor (paired odor: CS) paired with water (appetitive US) or a high concentration (20%) of sodium chloride solution (aversive US) and another odor (unpaired odor) without pairing with US five or six times each with pseudo-random sequences and with 5-min intervals. Retention was tested at 1 day after training. For pharmacology, 3 μL of saline or saline containing 2 μM epinastine or 200 μM flupentixol was injected into the hemolymph at 20 min before training.

We used differential appetitive or aversive conditioning procedures with water reward or sodium chloride punishment. In differential appetitive conditioning, individual animals received five trials, in which one of the two odors (peppermint and apple odors) was paired with water (unconditioned stimulus, US) and another odor was presented alone without pairing with US with an inter-trial interval (ITI) of 5 min (Figure 1). We refer to the former odor as paired odor (or conditioned stimulus, CS) and the latter odor as unpaired odor. Hypodermic syringes of 1 ml each were used for conditioning. A small filter paper was attached to the needle of the syringe. The syringe was filled with water, and the filter paper was soaked in peppermint or apple essence. For odor presentation, the filter paper was placed within 1 cm of the cricket's head. At 3 s after the onset of odor presentation, a drop of water was given to the mouth of the cricket for 2 s. The presence or absence of an MER was recorded during the first 3 s of odor presentation. In differential aversive conditioning experiment, individual animals received six trials, in which one of the two odors (vanilla and maple odors) was paired with a high concentration (20%) of sodium chloride solution (aversive US) and another odor was presented alone. Crickets exhibited MERs to these odors with high percentages (60–80%) prior to conditioning. The sequence of odor presentations was pseudo-randomized to avoid a possible sequential effect (Figure 1).

We also performed experiments using absolute appetitive conditioning procedures, in which an odor is paired with water reward, to facilitate comparison with results of our previous studies using absolute conditioning procedures (Unoki et al., 2005; Mizunami et al., 2009). The methods and the results of absolute appetitive conditioning are described in Supplementary Figures S3–S5.

### Retention test

For evaluation of retention performance, the rates of MER to the paired odor and the unpaired odor were compared (Figure 1) at 1 day after conditioning. After the last odor presentation in the test, a drop of water was presented to the mouth or an antenna and the resulting MER was tested. Crickets that did not exhibit an MER to water US were not used for data evaluation. Crickets discarded by this criterion were less than 3% in total.

### Pharmacology

For pharmacology, saline containing flupenthixol (Mustard et al., 2003) or epinastine (Roeder et al., 1998) was injected into the head haemolymph at 20 min before the start of training (Unoki et al., 2005). These drugs were purchased from Sigma-Aldrich (Tokyo, Japan).

### Data analysis

Occurrence of MER to odor presentation was measured during acquisition and in retention tests. In all experiments, percentage of MER (%MER) was calculated as the number of crickets that exhibited MER to the CS in the total number of crickets studied. Cochrans *Q*-test was used for within-group comparison of %MER during acquisition. McNemar's test was used for pairwise comparison of %MER between the odor paired with the US and the odor presented alone in acquisition. In the retention test, McNemar's test was used to compare %MER to the paired odor and the unpaired odor.

## Results

### Maxillary palpi extension response of crickets

**Figure 2:**
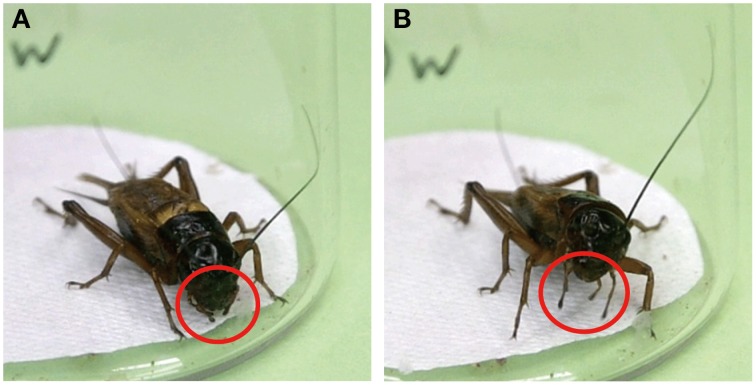
**Maxillary palpi extension response (MER) of the cricket**. **(A)** When a cricket is stationary, its maxillary palpi are typically held loosely beneath the mouthparts (red circles). **(B)** Upon presenting a drop of water to an antenna of the cricket, the cricket extended (red circles) and vigorously swung its maxillary palpi while raising its head.

Maxillary palpi of crickets are equipped with a number of olfactory receptors, contact chemoreceptors and mechanoreceptors, and crickets use these receptors to locate nearby food or water sources (Klein, 1981). When crickets are stationary, their maxillary palpi are usually held loosely beneath the mouthpart (Figure 2A). Upon application of water to the antennae, crickets extended and vigorously swung their maxillary palpi (Figure 2B). This response, which we term maxillary palpi extension response (MER), accompanied raising of the head and extension and swinging of the labial palpi and was immediately followed by vigorous swinging of the antennae, protraction of the mouth forward and upward, and frequent initiation of locomotor actions (Supplementary Movie 1). In short, MER is an initial phase of exploratory behavior in search for water or odor source.

We observed that crickets often extended and vigorously swung their maxillary palpi when a small piece of filter paper soaked with an essence of some odors, such as vanilla and maple odors, was presented near the antennae. In contrast, presentation of other odors, such as peppermint odor and apple odor, rarely induced MER (10% or less). In subsequent experiments, peppermint and apple odors were used for appetitive conditioning with water reward, while vanilla and maple odors were used for aversive conditioning with sodium chloride punishment.

### Acquisition and retention in differential aversive MER conditioning

**Figure 3:**
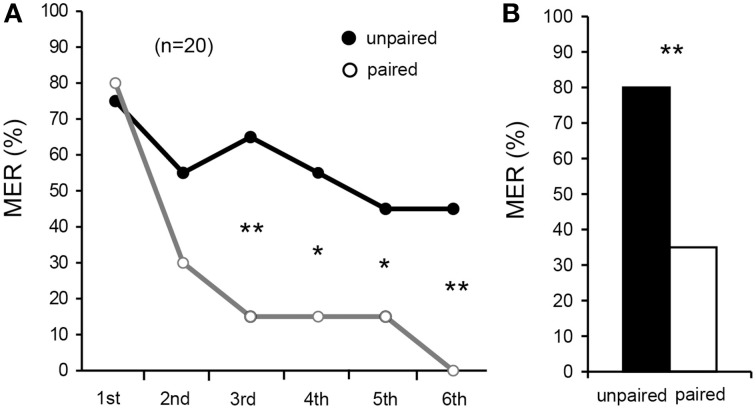
**Differential aversive olfactory conditioning of MER with sodium chloride US**. **(A)** Acquisition performance. Percentages of MER to an odor paired with 20% sodium chloride solution (paired odor, open circle) and those to an odor presented alone (unpaired odor, filled circle) in each conditioning trial are shown. Crickets were subjected to six pairing trials to associate an odor with sodium chloride solution (aversive US) and to presentation of another odor without pairing with the US with 5-min intervals, and percentages of MERs to the paired odor (gray graph) and the unpaired odor (black graph) were compared. The number of animals tested is shown in parentheses. **(B)** Retention performance at 1-day after conditioning. Percentages of MERs to the paired odor (white bar) and the unpaired odor (black bar) are shown. Percentage of MER to the paired odor was significantly lower than that to the unpaired odor. ^*^p < 0.05; ^**^p < 0.01.

We first attempted to establish a procedure for aversive olfactory conditioning of MER. We used a high concentration (20%) of sodium chloride solution as aversive US and vanilla and maple odors as CSs. We observed that repeated presentation of these odors alone without pairing with US led to a slight decrease of %MER to the odor (see Figure 3A). Therefore, in order to discriminate pairing-specific decrement of %MER (associative conditioning effect) from this non-associative effect (habituation), we used a differential conditioning procedure in which one of the two odors (vanilla and maple odors) was paired with US (paired odor; CS) and another odor was presented alone (unpaired odor) to allow comparison of MERs to the CS and the unpaired odor. The odors were presented six times each in a pseudo-random sequence and with 5-min ITIs. Percentages of MER to vanilla odor and those to maple odor were similarly high (>70%) in the first trials, and acquisition and retention performances of the vanilla CS group did not differ from those of the maple CS group (Supplementary Figure S1). Therefore, data from the two subgroups, vanilla CS group and maple CS group, were pooled. Percentage of MER to the CS significantly decreased with increase in the number of trials (Figure 3A, Cochran's *Q*-test: χ^2^ = 38, *df* = 5, *p* = 0.00000032). For the unpaired odor, we observed a slight decrease of %MER by repeated presentation of the odor, although the difference was not statistically significant (Cochran's *Q*-test: χ^2^ = 5.1, *df* = 5, *p* = 0.41). Percentages of MER to the CS did not significantly differ from that to the unpaired odor in the 1st trial (McNemar's test: χ^2^ = 0.0, *df* = 1, *p* = 1.0) and in the 2nd trial (χ^2^ = 2.3, *p* = 0.13), but it was significantly greater than that to the unpaired odor in the 3rd (χ^2^ = 6.8, *p* = 0.0094), 4th (χ^2^ = 4.9, *p* = 0.027), 5th (χ^2^ = 4.2, *p* = 0.041), and 6th trials (χ^2^ = 7.1, *p* = 0.0077). Thus, we conclude that the decrease of %MER to the paired odor is pairing-specific.

Next, retention performance was tested at 1 day (24 h) after six-trial aversive conditioning. Aversively conditioned crickets exhibited a significantly lower %MER to the CS (paired odor) than to the unpaired odor (Figure 3B, McNemar's test: χ^2^ = 7.1, *df* = 1, *p* = 0.0077). Thus, 1-day aversive memory is in large part pairing-specific.

### Acquisition and retention in differential appetitive MER conditioning

**Figure 4:**
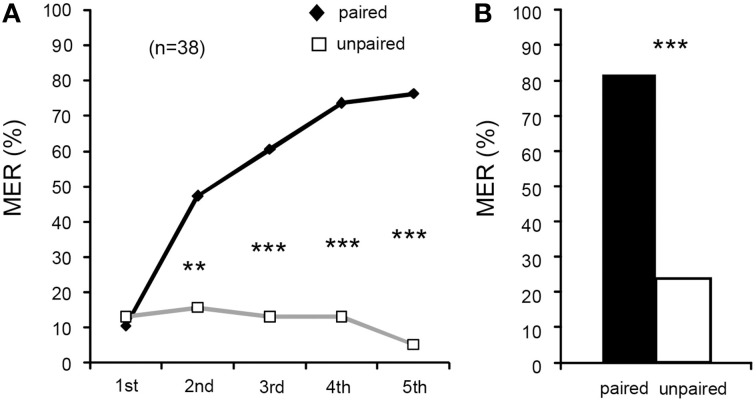
**Differential appetitive olfactory conditioning of MER with water US**. **(A)** Acquisition performance. Percentages of MER to the odor paired with water (paired odor: black graph) and the odor presented alone (unpaired odor; gray graph) are shown. Animals were subjected to five pairing trials to associate an odor with water and to presentation of another odor without pairing with the US with 5-min intervals. The number of animals tested is shown in parentheses. **(B)** Retention performance at 1 day after conditioning. Percentages of MERs to the paired odor (black bar) and those to the unpaired odor (white bar) are shown. The %MER to the paired odor was significantly higher than that to the unpaired odor. ^**^p < 0.01; ^***^p < 0.001.

We next attempted to establish a procedure for appetitive conditioning with water reward. In one group of crickets, one of two odors (peppermint and apple odors) was paired with water and the other odor was presented alone without pairing with US for five times each in a pseudo-random sequence and with 5-min ITIs (Figure 1). Because acquisition and 1-day retention performances in the peppermint CS group did not differ from those in the apple CS group (Supplementary Figure S2), data from the two sub-groups were pooled. In the first trial, %MER to either peppermint or apple odor was low (< 15%), and it significantly increased with increase in the number of trials (Figure 4A, Cochran's *Q*-test: χ^2^ = 53, *df* = 4, *p* = 0.000000000086). For the unpaired odor, we observed a slight decrease of %MER by repeated presentation of the odor, although the difference was not statistically significant (Cochran's *Q*-test: χ^2^ = 2.4, *df* = 4, *p* = 0.67). The %MER to the paired odor did not significantly differ from that to the unpaired odor in the 1st trial (McNemar's test: χ^2^ = 0, *df* = 1, *p* = 1), but it was significantly greater than that to the unpaired odor in the 2nd (χ^2^ = 8.6, *p* = 0.0033), 3rd (χ^2^ = 14, *p* = 0.00014), 4th (χ^2^ = 18, *p* = 0.000023), and 5th trials (χ^2^ = 25, *p* = 0.00000056). Thus, we conclude that the increase of %MER to the paired odor is pairing-specific.

Retention performance was tested at 1 day (24 h) after conditioning. The group that received differential appetitive conditioning exhibited a significantly larger %MER to the paired odor than to the unpaired odor (Figure 4B, McNemar's test: χ^2^ = 20, *df* = 1, *p* = 0.0000076). Thus, 1-day appetitive memory is in large part pairing-specific.

We also attempted to establish a procedure of absolute appetitive olfactory conditioning of the MER, in which an odor was paired with water reward (Supplementary Figures S3, S4). This was to facilitate comparison with results of our previous studies using a dual-choice preference test, in which we used absolute conditioning procedure (Matsumoto and Mizunami, 2002; Unoki et al., 2005; Mizunami et al., 2009). We found that a few pairing trials of absolute appetitive conditioning are sufficient to achieve pairing-specific associative effect and that 30-min or 1 day memory is in large part CS-specific (Supplementary Figure S4), in agreement with our findings using a dual-choice preference test.

### Epinastine, but not flupenthixol, impairs acquisition of differential appetitive MER conditioning

We then proceeded to a study on the effects of dopamine and octopamine receptor antagonists on appetitive and aversive learning using differential procedures. We used epinastine (Roeder et al., 1998) and flupenthixol (Mustard et al., 2003), potent antagonists of insect octopamine and dopamine receptors, respectively, since they were the most effective drugs to impair appetitive and aversive learning among drugs we used in our studies in crickets (Unoki et al., 2005, 2006; Mizunami et al., 2009; Nakatani et al., 2009; Matsumoto et al., 2013).

**Figure 5:**
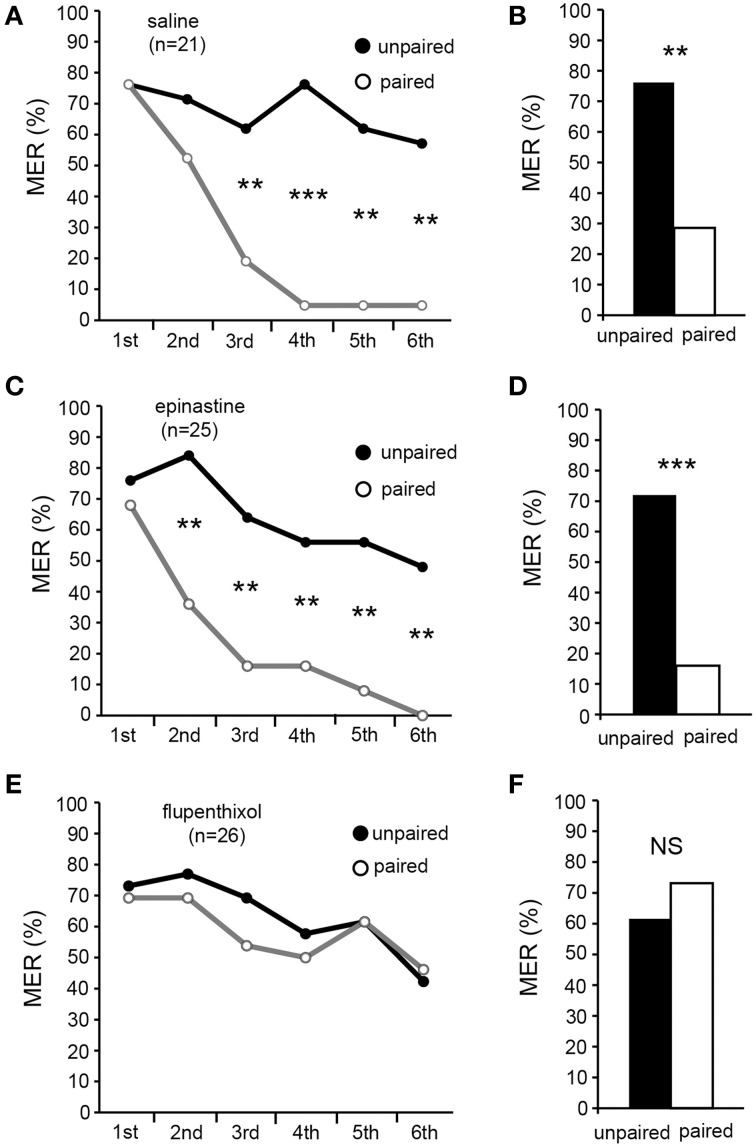
**Epinastine, but not flupenthixol, impairs acquisition of appetitive MER conditioning**. At 20 min prior to differential appetitive conditioning, crickets in three groups were each injected with 3 μl of saline (saline group, **A,B**) or saline containing 2 μM epinastine (epinastine group, **C,D**) or 200 μM flupenthixol (flupenthixol group, **E,F**). **(A,C,E)** Acquisition performance of the saline group **(A)**, epinastine group **(C)** and flupenthixol group **(E)**. Percentages of MER to the paired odor (black graphs) and those to the unpaired odor (gray graphs) in each conditioning trial are shown. The saline group and flupenthixol group exhibited effective acquisition, but the epinastine group did not. **(B,D,F)** One-day retention performance of the saline group **(B)**, epinastine group **(D)**, and flupenthixol group **(F)**. Percentages of MER to the paired odor and those to the unpaired odors are shown. The saline group and flupenthixol group exhibited significantly higher %MER to the CS (black bars) than to the unpaired odor (white bars), indicating that the memory is CS-specific. In contrast, in the epinastine group, percentage of MER to the CS was as low as that to the unpaired odor, indicating no CS-specific memory. ^***^p < 0.001; ^**^p < 0.01; NS: non-significant (*p* > 0.05).

We first tested the effects of epinastine and flupentixol on differential MER conditioning with water reward. Three groups of crickets were each injected with 3 μl of saline (saline group) or saline containing 2 μM epinastine (epinastine group) or 200 μM flupenthixol (flupenthixol group) at 20 min prior to 5 trials of appetitive conditioning. The doses and the timing of injection were based on previous studies (Unoki et al., 2005, 2006; Mizunami et al., 2009; Nakatani et al., 2009). The saline and flupenthixol groups exhibited significant increases in %MER to the CS with progress of training (Figures 5A,E, Cochran's *Q*-test: saline: χ^2^ = 36, *df* = 4, *p* = 0.00000025; flupenthixol: χ^2^ = 46, *p* = 0.0000000022), but the epinastine group did not (Figure 5C, Cochran's *Q*-test: χ^2^ = 8.5, *df* = 4, *p* = 0.074). In these three groups, %MER to the unpaired odor, on the other hand, did not change with progress of training (Figures 5A,C,E, Cochran's *Q*-test: saline: χ2 = 4.82, *df* = 4, *p* = 0.31; epinastine: χ2 = 0.76, *p* = 0.94; flupenthixol: χ2 = 3.0, *p* = 0.55). The saline and flupenthixol groups exhibited significantly higher %MER to the CS than to the unpaired odor in the 3rd (Figures 5A,E, McNemar's test: saline: χ2 = 8.6, *df* = 1, *p* = 0.0033; flupenthixol: χ2 = 12, *p* = 0.00069), 4th (saline: χ2 = 12, *p* = 0.00051; flupenthixol: χ2 = 14, *p* = 0.00018), and 5th trials (saline: χ2 = 13, *p* = 0.00030; flupenthixol: χ2 = 20, *p* = 0.0000076). However, %MER to appetitive CS in the epinastine group did not significantly differ from that to the unpaired odor in all trials (Figure 5C, McNemar's test: *p* > 0.05). The results indicate that epinastine, but not flupenthixol, impairs acquisition of appetitive learning with water reward. We thus suggest that octopamine, but not dopamine, participates in acquisition of appetitive learning.

Retention performance was tested at 1 day (24 h) after conditioning. The saline and flupenthixol groups exhibited a high %MER to the CS (>60%), and it was significantly greater than that to the unpaired odor (Figures 5B,F, McNemar's test: saline: χ^2^ = 12, *df* = 1, *p* = 0.00051; flupenthixol: χ^2^ = 21, *p* = 0.0000044). In the epinastine group, on the other hand, %MER to the CS was low (< 40%) and it did not significantly differ from that to the unpaired odor (Figure 5D, χ^2^ = 0.25, *p* = 0.62). Thus, the epinastine group exhibited no CS-specific memory, whereas 1-day retention of CS-specific memory was intact in the flupenthixol group as in the saline group.

We also confirmed that epinastine, but not flupenthixol, impaired initial acquisition and 1-day retention using a five-trial absolute appetitive conditioning procedure (Supplementary Figure S5). Thus, we suggest that octopamine, but not dopamine, is required for acquisition of appetitive learning, regardless of the procedure being differential or absolute.

### Flupenthixol, but not epinastine, impairs acquisition of aversive MER conditioning

**Figure 6:**
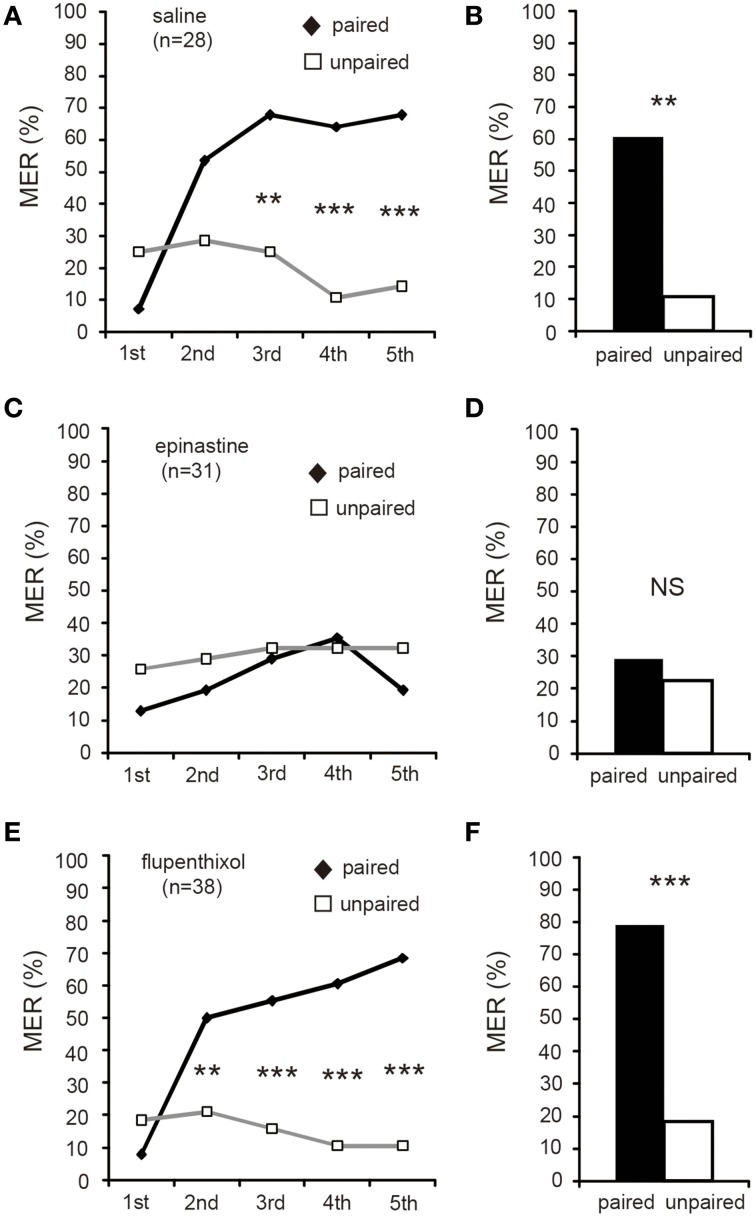
**Flupenthixol, but not epinastine, impairs acquisition of aversive MER conditioning**. At 20 min prior to differential aversive conditioning, crickets in three groups were each injected with 3 μl of saline (saline group, **A,B**) or saline containing 2 μM epinastine (epinastine group, **C,D**) or 200 μM flupenthixol (flupenthixol group, **E,F**). **(A,C,E)** Acquisition performance of the saline group **(A)**, epinastine group **(C)** and flupenthixol group **(E)**. Percentages of MER to the paired odor (gray graph) and those to the unpaired odor (black graphs) in each conditioning trial are shown. The saline and epinastine groups exhibited significantly lower %MER to the aversive CS (gray graph) than to the unpaired odor (black graph), but the flupenthixol group exhibited no significantly different %MER to the CS than that to the unpaired odor. **(B,D,F)** Retention performance at 1 day after conditioning of the saline group **(B)**, epinastine group **(D)** and flupenthixol group **(F)**. Percentages of MER to the paired odor and the unpaired odor are shown. The saline and epinastine groups exhibited significantly lower %MER to the aversive CS (white bars) than to the unpaired odor (black bars), indicating CS-specific aversive memory. In the flupenthixol group, on the other hand, %MER to the CS did not significantly differ from that to the unpaired odor, indicating no CS-specific memory. The number of animals tested is shown in parentheses.^**^p < 0.01; ^***^p < 0.001; NS: non-significant (*p* > 0.05).

In the final experiment, we tested the effects of epinastine and flupenthixol on differential MER conditioning with sodium chloride punishment. Three groups of crickets were each subjected to injection of 3 μl saline (saline group) or saline containing 2 μM epinastine (epinastine group) or 200 μM flupenthixol (flupenthixol group) at 20 min before 6 trials of differential aversive conditioning. In the saline and epinastine groups, %MER to aversively conditioned odor (CS, paired) significantly decreased with progress of training (Figures 6A,C, Cochran's *Q*-test: saline: χ^2^ = 42, *df* = 5, *p* = 0.000000048; epinastine: χ^2^ = 39, *p* = 0.00000021). Percentages of MER to the unpaired odor slightly decreased with progress of training, but the difference was not statistically significant (Cochran's *Q*-test: saline: χ^2^ = 5.0, *df* = 5, *p* = 0.42; epinastine: χ^2^ = 10, *p* = 0.076). The saline and epinastine groups exhibited significantly lower %MER to the CS than to the unpaired odor in the 3rd (Figures 6A,C, McNemar's test: saline: χ^2^ = 7.1, *df* = 1, *p* = 0.0077; epinastine: χ^2^ = 10, *p* = 0.0015), 4th (saline: χ^2^ = 13, *p* = 0.00030; epinastine: χ^2^ = 6.8, *p* = 0.0094), 5th (saline: χ^2^ = 10, *p* = 0.0015; epinastine: χ^2^ = 10, *p* = 0.0015), and 6th trials (saline: χ^2^ = 9.1, *p* = 0.0026; epinastine: χ^2^ = 10, *p* = 0.0015). In the flupenthixol group, on the other hand, there were no significant changes of %MER not only to the control odor (unpaired odor) but also to aversive CS with training (Figure 6E, Cochran's *Q*-test: to CS: χ^2^ = 5.0, *df* = 5, *p* = 0.42; to the unpaired odor: χ^2^ = 7.9, *p* = 0.16). The %MER to aversive CS in this group did not significantly differ from that to the unpaired odor in all trials (McNemar's test: *p* > 0.05). The results indicate that flupenthixol, but not epinastine, impairs acquisition of aversive learning with sodium chloride US. We thus suggest that dopamine, but not octopamine, is required for acquisition of aversive learning.

In a 1-day retention test, the saline and epinastine groups exhibited significantly lower %MER to the aversive CS than to the unpaired odor (Figures 6B,D, McNemar's test: saline: χ^2^ = 8.1, *df* = 1, *p* = 0.0044; epinastine: χ^2^ = 12, *p* = 0.0005). In the flupenthixol group, on the other hand, %MER to the aversive CS did not significantly differ from that to the unpaired odor (Figure 6F, χ^2^ = 0.36, *p* = 0.55). Thus, the epinastine group exhibited CS-specific memory as did the saline group, but the flupenthixol group did not.

## Discussion

We showed that olfactory conditioning of MER provides an excellent paradigm to investigate acquisition performance of appetitive and aversive conditioning in crickets. MER is an initial phase of exploratory movements in search for water or odor sources, and the occurrence of MER to an odor can be easily judged if crickets and their palpi are stationary prior to water or odor presentation. Crickets exhibited high percentages of MER to some odors but not to other odors, and we used the former odors in aversive conditioning and the latter odors in appetitive conditioning. We observed that repeated presentation of one of the former odors alone led to a slight decrease of the MER, although the difference was not statistically significant. We thus used a differential aversive conditioning procedure for evaluation of the pairing-specific effect. In contrast, we observed that repeated presentation of one of the latter odors alone did not lead to a change of the MER. In appetitive or aversive conditioning, we observed a significant difference in the MER between the paired and unpaired odors in the 2nd or 3rd trial. Thus, only a few trials are sufficient to induce a conditioning effect. Retention tests performed 1 day after appetitive or aversive conditioning demonstrated that the memory is in large part CS-specific. Olfactory MER conditioning thus provides a robust measure of acquisition and retention in olfactory learning in crickets.

In addition to differential conditioning procedures used in this study, we established absolute appetite conditioning procedure to associate an odor with water reward (Supplementary Figure S3), and we observed that only a few trials are sufficient to induce a conditioning effect (Supplementary Figure S4A) and memory at 30 min or 1 day after 5-trial absolute appetitive conditioning is in large part CS-specific (Supplementary Figure S4B). Therefore, both of the differential and absolute olfactory MER conditioning procedures provide robust measures of acquisition and retention in olfactory learning in crickets.

We now have two procedures to test the effect of olfactory conditioning training, the MER test and the operant dual-choice preference test. The former is useful for studying mechanistic aspects of learning and memory, while the latter provides insights into the behavioral significance of them. Crickets should open up further possibilities for studying mechanistic and adaptive aspects of learning and memory.

### Comparison of MER conditioning with conditioning of feeding responses in other insects

Conditioning of MER by pairing an odor with water reward described here is analogous to olfactory conditioning of mouthpart movements reported in several insects, especially that of proboscis extension response (PER) in honey bees (Giurfa and Sandoz, 2012). We observed 60–80% MERs to an odor paired with water US in crickets after four pairing trials, the percentages being slightly less than those of PERs to sucrose-associated odor in honey bees (more than 80% after three or more trials; Menzel and Giurfa, 2006; Matsumoto et al., 2014) and being comparable or slightly higher than those of PERs in the moth *Manduca sexta* (50–70%; Daly and Smith, 2000), those of maxilla-labium extension responses in *Camponotus* ants (40–60%; Guerrieri et al., 2011) and those of maxillary palpi opening reactions in the desert locust *Schistocerca gregaria* (40–50%; Simões et al., 2011). In all of those studies, the conditioned response (mouthpart movement) was measured in harnessed insects, but we measured it from freely moving animals placed in a small beaker in this study. We found that harnessed crickets also exhibit MER, but the percentage of MER was not as high as that in crickets placed in a beaker (data not shown).

We were able to evaluate aversive conditioning by a decrease of the MER to odors paired with sodium chloride punishment. This allows evaluation of appetitive conditioning and aversive conditioning in very similar experimental conditions and thus facilitates comparisons of the neural basis and molecular basis of acquisition in appetitive and aversive conditioning. For future studies, it will be more ideal to find odors that produce MER at a rate of about 50% and use them in both appetitive and aversive conditioning. To our knowledge, little effort has been directed toward establishing a procedure to use the decrease in the rate of feeding response for evaluation of aversive conditioning in other species of insects.

### Roles of octopamine and dopamine in appetitive and aversive conditioning

We found that octopamine and dopamine receptor antagonists specifically impair acquisition of appetitive learning with water reward and aversive learning with sodium chloride punishment, respectively (Figures 5, 6, Supplementary Figure S5). It should be pointed out that the experimental procedures for appetitive and aversive learning were not exactly the same, namely, the odors used as CS and the number of conditioning trials differed. Therefore, whether such differences might account for different effects of antagonists should be discussed. This possibility, however, is unlikely since we observed that the effects of antagonists were conserved in experiments with different kinds of CS and different number of trials (Unoki et al., 2005, 2006; Mizunami et al., 2009; Nakatani et al., 2009; Mizunami and Matsumoto, 2010). Therefore, we suggest that octopamine and dopamine mediate reinforcement signals for acquisition of appetitive and aversive learning, respectively, in crickets. In accordance with our suggestion, it has been suggested in honey bees that octopamine participates in appetitive learning with sucrose reward (Hammer and Menzel, 1998) and that dopamine participates in aversive learning with electric shock punishment (Vergoz et al., 2007).

Based on results of recent studies on fruit-flies, however, it was concluded that different sets of dopamine neurons mediate appetitive and aversive reinforcements in olfactory conditioning with sucrose, water, or electric shock, while octopamine neurons convey sweet taste signals of sucrose reward to dopamine neurons (Burke et al., 2012; Liu et al., 2012; Lin et al., 2014; Yamagata et al., 2015: For an alternative view, see Kim et al., 2013.). Thus, we suggest that neurotransmitters mediating appetitive reinforcement differ in crickets and fruit-flies, at least in learning with water reward, whereas those mediating aversive reinforcement are the same. In order to confirm that octopamine and dopamine mediate appetitive and aversive reinforcement, respectively, we are currently performing studies using RNAi (Shinmyo et al., 2006; Takahashi et al., 2009) and CRISPR/Cas9 systems (Cong et al., 2013; Mali et al., 2013) to knockdown or knockout expression of octopamine and dopamine receptor genes in crickets.

Concerning signals that animals use for reinforcement, we recently obtained evidence that the prediction error theory, which states that the discrepancy, or error, between actual reward and predicted reward determines whether learning occurs (Rescorla and Wagner, 1972), is applicable to crickets (Terao et al., 2015). This theory is known as the best theory to account for associative learning in mammals (Schultz, 2006, 2013) and there is strong evidence that dopamine neurons in the midbrain mediate appetitive prediction error signals (Schultz, 2013), although whether they also mediate aversive prediction error signals is controversial (Matsumoto and Hikosaka, 2009; Fiorillo, 2013). For crickets, we reported results of pharmacological analysis suggesting that octopamine neurons mediate appetitive prediction error signals (Terao et al., 2015), although it remains to be determined whether dopamine neurons mediate aversive prediction error signals in crickets. Future electrophysiological studies on dopamine and octopamine neurons projecting to the mushroom body, a higher-order associative center participating in olfactory learning (Menzel and Giurfa, 2006; Davis, 2011; Menzel, 2012), are needed to clarify neural mechanisms of prediction error computation in associative learning in crickets.

In conclusion, we suggest that there is an unexpected diversity in neurotransmitters mediating appetitive reinforcement, while those mediating aversive reinforcement are conserved, among different species of insects. There arises a question of to what extent the neurotransmitter mechanisms of learning differ between different insect species and how such diversity has evolved (see also Mizunami et al., 2015). Further studies on a number of insects, such as honey bees, moths, and cockroaches, are needed for answering this interesting question.

## Author contributions

YM designed and carried out experiments, analyzed and interpreted data, and wrote the manuscript. CM, RW, and SI carried out experiments and analyzed and interpreted data. MM designed experiments, analyzed and interpreted data, and wrote the manuscript.

### Conflict of interest statement

The authors declare that the research was conducted in the absence of any commercial or financial relationships that could be construed as a potential conflict of interest.
