# Supplementary material for: Roles of octopamine and dopamine in appetitive and aversive memory acquisition studied in olfactory conditioning of maxillary palpi extension response in crickets
Source: Front Behav Neurosci. 2015 Sep 1;9:230. doi: 10.3389/fnbeh.2015.00230 (PMC4555048; doi:10.3389/fnbeh.2015.00230)
Supplement: Supplementary file 1 [file Presentation1.PDF]

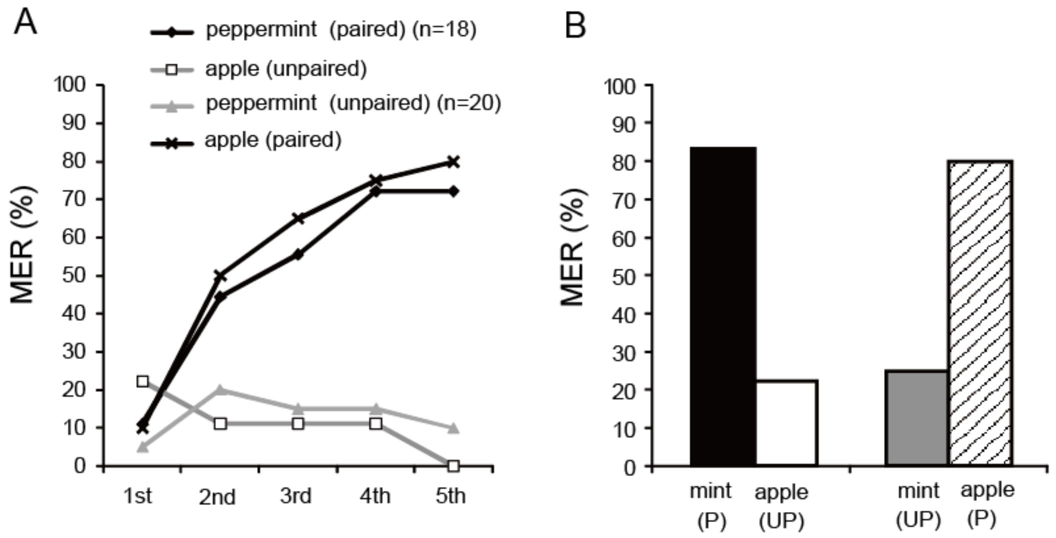

FIGURE S1. No odor bias in differential aversive MER conditioning with maple CS and vanilla CS. (**A**) Acquisition performance in 6-trial differential aversive conditioning in groups with maple CS and that with vanilla CS. Percentages of MER to the maple or vanilla odors, one of which was paired with sodium chloride solution (paired odor) and the other of which was presented alone (unpaired odor), are shown. The number of animals tested is shown in parentheses. (**B**) Retention performance at 1 day after 6-trial differential aversive conditioning of the group with vanilla CS (left) and maple CS (right). Percentages of MERs to maple or vanilla odor used as the paired (P) odor (CS) or the unpaired (UP) odor are shown. The acquisition performance and retention performance of the maple CS group were very similar to those of the vanilla CS group. Thus, the data from the two groups were pooled.
