# Supplementary material for: Roles of octopamine and dopamine in appetitive and aversive memory acquisition studied in olfactory conditioning of maxillary palpi extension response in crickets
Source: Front Behav Neurosci. 2015 Sep 1;9:230. doi: 10.3389/fnbeh.2015.00230 (PMC4555048; doi:10.3389/fnbeh.2015.00230)
Supplement: Supplementary file 2 [file Presentation2.PDF]

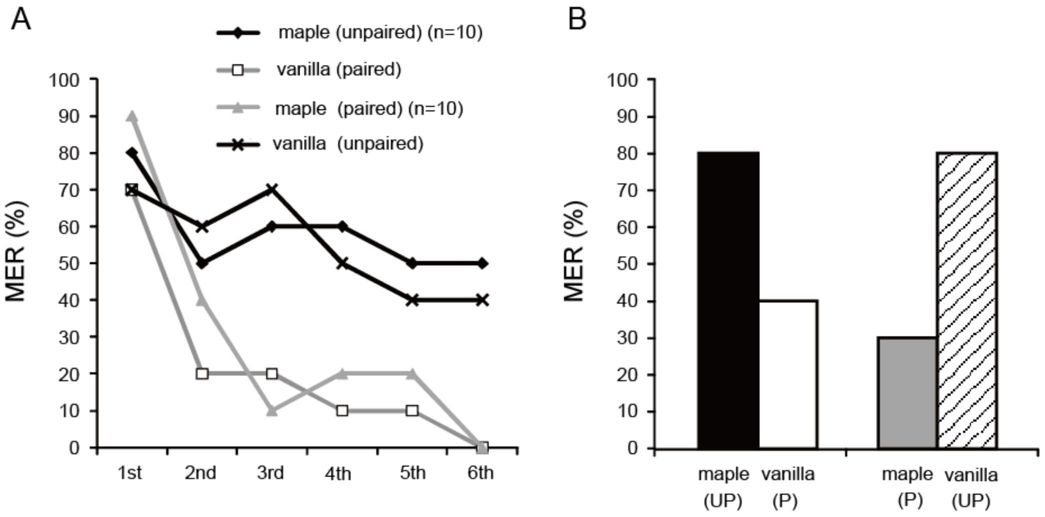

FIGURE S2. No odor bias in differential appetitive MER conditioning with peppermint CS and apple CS. (**A**) Acquisition performance of differential appetitive conditioning in the peppermint CS group and the apple CS group. Percentages of MERs to the peppermint and apple odors, one of which was paired with US (paired) and the other of which was presented alone (unpaired), are shown. The groups were subjected to 5 trials to associate peppermint or apple odor with water reward. (**B**) Retention performance at 1 day (24 h) after differential absolute conditioning in groups with the peppermint CS (left) and the apple CS (right). Percentages of MERs to the peppermint or apple odor used as the paired (P) odor (CS) or the unpaired (UP) odor are shown. The number of animals tested is shown in parentheses. The acquisition performance and retention performance of the peppermint CS group were very similar to those of the apple CS group. Thus, data from the two groups were pooled.
