# Supplementary material for: Roles of octopamine and dopamine in appetitive and aversive memory acquisition studied in olfactory conditioning of maxillary palpi extension response in crickets
Source: Front Behav Neurosci. 2015 Sep 1;9:230. doi: 10.3389/fnbeh.2015.00230 (PMC4555048; doi:10.3389/fnbeh.2015.00230)
Supplement: Supplementary file 3 [file Presentation3.PDF]

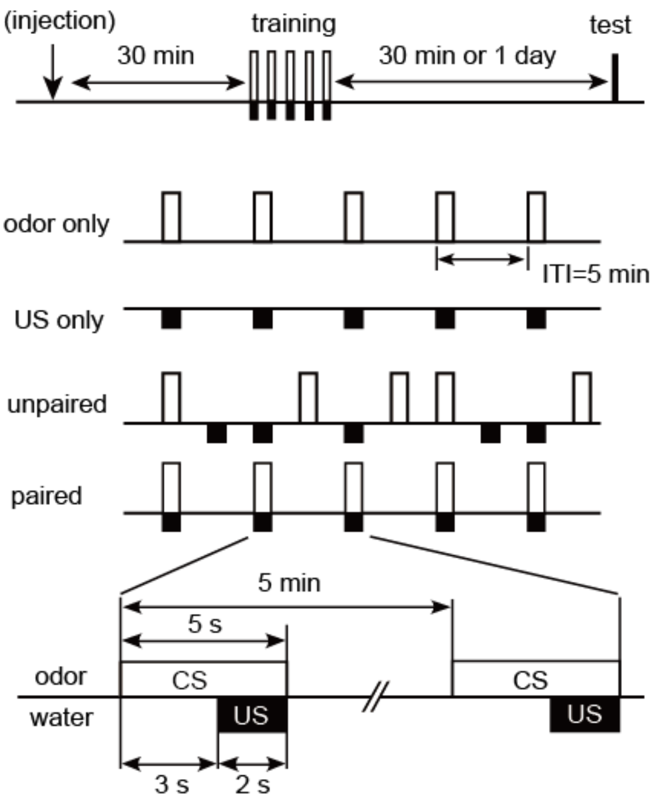

FIGURE S3. Procedures for absolute appetitive olfactory conditioning of MER with water reward. In absolute appetitive conditioning with water reward, the paired group was subjected to five conditioning trials to associate an odor (CS) with water (US) with an inter-trial interval (ITI) of 5 min. The unpaired group was subjected to unpaired presentations of CS and US five times each with pseudo-random sequences and with 2.5-min intervals. The CS only group was presented with CS without pairing with US five times with 5-min intervals. The US only group was presented with US five times with 5-min intervals. For evaluation of retention performance, %MER to the odor presented in training was compared to that to an odor not used in training (a novel odor) in the paired, unpaired and CS-only groups. In the US only group, the rates of MERs to two novel odors were compared. Crickets were presented with odors for 5 sec each with 5-min intervals, and the presence or absence of an MER was recorded. Retention was tested at 30 min or 1 day after training. For pharmacology, 3μl of saline or saline containing 2 μM epinastine or 200 μM flupentixol was injected at 30 min before training.
