# Supplementary material for: Roles of octopamine and dopamine in appetitive and aversive memory acquisition studied in olfactory conditioning of maxillary palpi extension response in crickets
Source: Front Behav Neurosci. 2015 Sep 1;9:230. doi: 10.3389/fnbeh.2015.00230 (PMC4555048; doi:10.3389/fnbeh.2015.00230)
Supplement: Supplementary file 4 [file Presentation4.PDF]

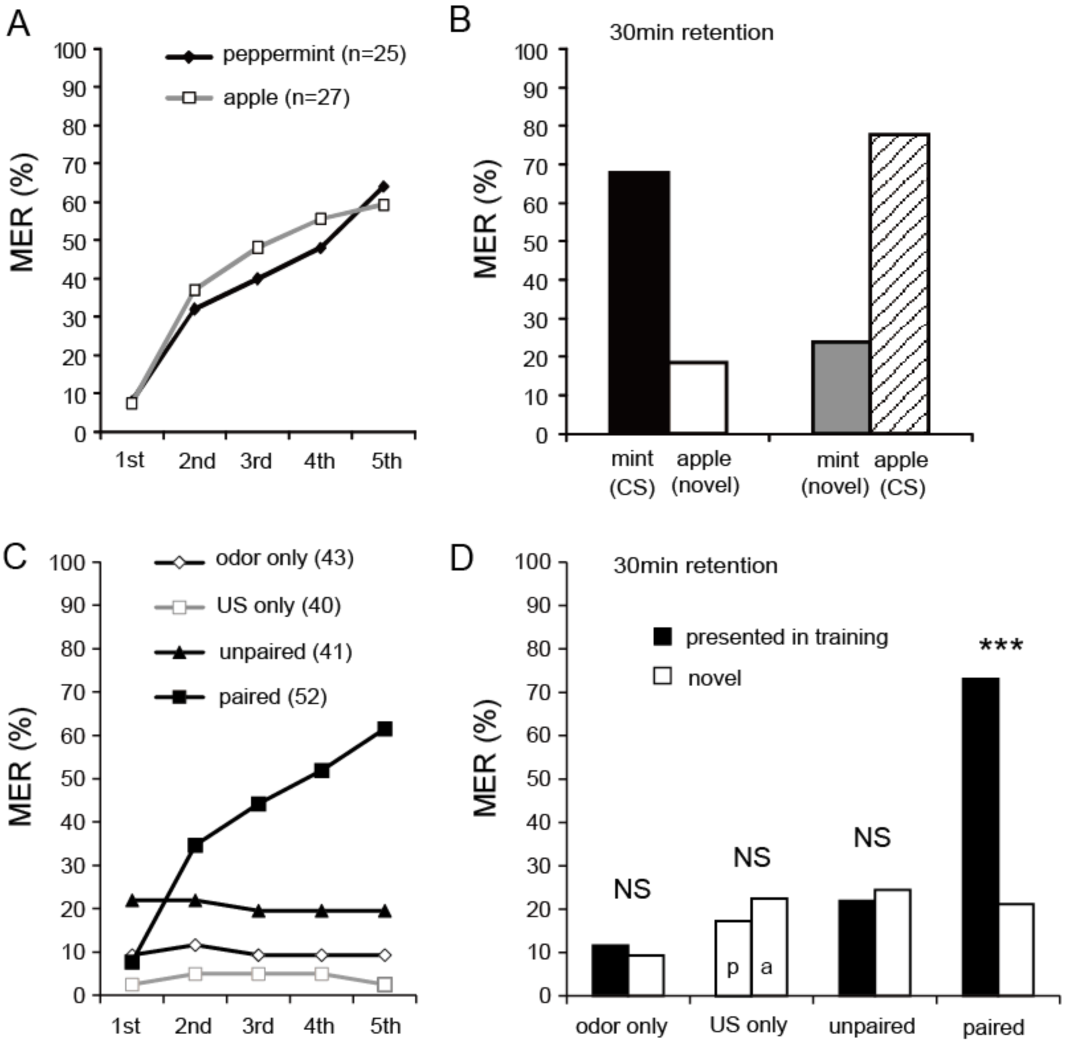

FIGURE S4. ACQUISITION AND RETENTION IN ABSOLUTE APPETITIVE CONDITIONING OF MER. We established an absolute conditioning procedure with water reward. (**A**, **B**) Acquisition performance (**A**) and 30-min retention performance (**B**) of a group that recieved 5 conditioning trials to associate peppermint or apple odor (CS) with water US with an inter-trial interval (ITI) of 5 min. The percentage of MER (%MER) during a 3-sec period of CS presentation prior to US presentation (see **Figure S3**) is shown. Because both initial acquisition and 30-min retention performances in the group in which peppermint odor was used as CS (peppermint CS group) did not differ from those in which apple odor was used as CS (apple CS group), data from the two sub-groups were pooled and designated as the paired group. (**C**) Acquisition performance of the paired group and three control groups. The %MER to the peppermint or apple odor of the paired group was less than 10% in the first trial (i.e., immediately before the first CS-US pairing trial, see **Figure S3**), but it increased to more than 60% in the fifth trial (after the fourth trial). The increase in %MER to the CS with an increase in the number of trials was statistically significant (**C**, Cochran's Q test: χ^2 = 50,

df = 4, p = 0.00000000030). Another three control groups were each subjected to presentation of an odor alone without pairing with water US (odor only group), presentation of water alone (US only group) or unpaired presentation of CS and US (unpaired group) for 5 times each. The ITI was 5 min except for in the unpaired group, in which it was 2.5 min (see **Figure S3**). In all control groups, %MER was less than 25% in all trials and there was no significant increase of %MER with an increase of the number of trials (Cochran's Q test: odor only: χ^2 = 0.26, df = 4, p = 0.99; US only: χ^2 = 0.80, p = 0.94; unpaired: χ^2 = 0.21, p = 0.99; In the US only group, %MER at 3 sec prior to US presentation was calculated.). Comparison among the groups showed that %MER in the paired group did not significantly differ from the percentages in the unpaired groups in the 1st trial (Fisher's exact test adjusted by Holm's method: p > 0.05), but it was significantly higher than the percentages in the control groups in the 3rd, 4th and 5th trials (Fisher's exact test adjusted by Holm's method: 3rd: p < 0.05; 4th: p < 0.01; 5th: p < 0.001). We thus conclude that the increase of MER by training is pairing-specific and not due to non-associative effects. (**D**) 30-min retention performance after five-trial absolute appetitive conditioning of the paired group and three control groups. The %MERs to the odor presented in training and that to a novel odor in the paired group and non-associative control groups are shown. In the paired group, %MER to the CS and that to a novel odor were calculated once for each (When peppermint odor was the CS, apple odor was the novel odor and vice versa.). The paired group exhibited a high %MER to the CS (>70%) and it was significantly higher than that to the novel odor (McNemar's test: χ^2 = 23, df = 1, p =0.0000014). In the odor only group and the unpaired group, %MER to the odor presented in training was low (<30%) and it did not significantly differ from that to the novel odor (McNemar's test: odor only: χ^2 = 0, df = 1, p = 1.0; unpaired: χ^2 = 0, p = 1.0). In the US only group, in which MERs to two novel odors were tested, %MER was low (<30%) and did not significantly differ between the two odors (US only: χ^2 = 0.1, df = 1, p = 1.0). The results indicate that 30-min memory in the paired group is CS-specific and that there was no obvious memory in non-associative control groups.
