# Supplementary material for: Roles of octopamine and dopamine in appetitive and aversive memory acquisition studied in olfactory conditioning of maxillary palpi extension response in crickets
Source: Front Behav Neurosci. 2015 Sep 1;9:230. doi: 10.3389/fnbeh.2015.00230 (PMC4555048; doi:10.3389/fnbeh.2015.00230)
Supplement: Supplementary file 5 [file Presentation5.PDF]

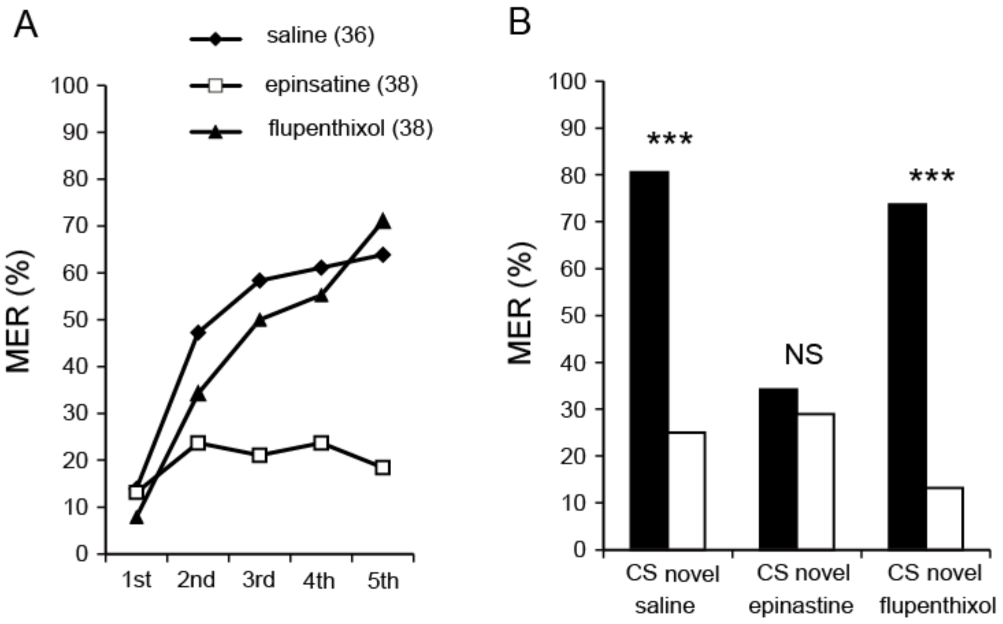

FIGURE S5. Epinastine, but not flupenthixol, impairs absolute appetitive MER conditioning. At 30 min prior to absolute appetitive conditioning, crickets in three groups were each injected with 3 μl of saline (saline group) or saline containing 2 μM epinastine (epinastine group) or 200 μM flupenthixol (flupenthixol group). (**A**) Acquisition performance. Percentages of MER to the paired odor in the saline group, epinastine group and flupenthixol group are shown. The saline and flupenthixol groups exhibited significant increases in percentages of MER to the CS with progress of training (Cochran's Q test: saline: χ^2 = 33, df = 4, p = 0.0000011; flupenthixol: χ^2 = 44, p = 0.0000000059), but the epinastine group did not (Cochran's Q test: χ^2 = 2.9, df = 4, p = 0.58). There was no significant difference in %MER among the three groups in the 1st and 2nd trials (Fisher's exact test adjusted by Holm's method, p > 0.05 for between-group comparison in the 1st and 2nd trials), but %MER in the epinastine group was significantly lower than that in the saline group in the 3rd, 4th and 5th trials (Fisher's exact test: 3rd: p = 0.0017; 4th: p = 0.0020; 5th: p = 0.0001). In contrast, %MER in the flupenthixol group did not significantly differ from that in the saline group in the 3rd, 4th and 5th trials (Fisher's exact test: 3rd: p = 0.49; 4th: p = 0.64; 5th: p = 0.62). The results indicate that epinastine, but not flupenthixol, impairs acquisition of appetitive learning. (**B**) Retention performance at 1 day (24 h) after conditioning. Percentages of MER to the CS and those to the novel odor in the saline group, epinastine group and flupenthixol group are shown. The saline and flupenthixol groups exhibited a high %MER to the CS (>70%), and it was significantly greater than that to the novel odor (McNemar's test: saline: χ^2 = 18, df = 1, p = 0.000022; flupenthixol: χ^2 = 21, p = 0.0000044). In the epinastine group,

on the other hand, %MER to the CS was low (<40%) and it did not significantly differ from that to the novel odor ($\chi^2 = 0.17$, $p = 0.68$). Thus, the epinastine group exhibited no CS-specific memory, whereas 1-day retention of CS-specific memory was intact in the flupenthixol group as in the saline group. The number of animals tested is shown in parentheses. ***: $p<0.001$; NS: non-significant ($p>0.05$).
